# A qualitative exploration of forensic pathology service staff perceptions of the implementation barriers and facilitators of manual- and electronic injury mortality surveillance system methods in South Africa

**DOI:** 10.1186/s12889-023-17337-5

**Published:** 2023-11-28

**Authors:** N. Arendse, Z. Goolam Nabi, A. van Niekerk

**Affiliations:** 1 Institute for Social and Health Sciences, University of South Africa (Unisa), Johannesburg, Gauteng South Africa; 2 Masculinity and Health Research Unit, South African Medical Research Council-Unisa, Cape Town, South Africa

**Keywords:** Injury mortality, Injury surveillance methodologies, Surveillance, National Injury Mortality Surveillance System (NIMSS), Surveillance system attributes

## Abstract

**Background:**

Injury mortality surveillance systems are critical to monitor changes in a population’s injury outcomes so that relevant injury prevention responses may be adopted. This is particularly the case in South Africa, where the injury burden is nearly twice the global rate. Regular evaluations of surveillance systems are pivotal to strengthening surveillance capacity, performance, and cost effectiveness. The National Injury Mortality Surveillance System (NIMSS) is an injury mortality surveillance system that is currently focused in Mpumalanga and utilises manual and electronic web-based systems for data collection. This study explored Forensic Pathology Service (FPS) staff perceptions of the implementation barriers and facilitators of manual- and electronic injury mortality surveillance system methods.

**Methods:**

A qualitative study was employed using purposive sampling. Forty-seven participants, aged 29 to 59 years comprising 31 males and 16 females were recruited across 21 FPS facilities that serve the province. The formative evaluation occurred over the November 2019 to November 2022 period. Twelve focus group discussions were thematically analysed to determine emerging themes and patterns related to the use of the system using the WHO surveillance system guidelines as a framework.

**Results:**

The key themes concerning the barriers and facilitators were located along WHO attributes of simplicity, acceptability, timeliness, flexibility, data quality and stability. Distinctions between the manual and e-surveillance systems were drawn upon across the attributes highlighting their experience with the system, user preference, and its contextual relevance. With Mpumalanga predominantly rural, internet connectivity was a common issue, with most participants consequently showing a preference for the manual system, even though the electronic system’s automated internal validation process was of benefit. The data quality however remained similar for both methods. With program stability and flexibility, the manual system proved more beneficial as the dataset was reported to be easily transferrable across computer devices.

**Conclusion:**

Obtaining FPS perceptions of their experiences with the system methodologies are pertinent for the enhancement of injury surveillance systems so to improve prospective engagements with the systems. This will facilitate timely and accurate injury mortality information which is vital to inform public policy, and injury control and prevention responses.

**Supplementary Information:**

The online version contains supplementary material available at 10.1186/s12889-023-17337-5.

## Background

Injuries continue to be a public health concern accounting for an estimated 4.4 million deaths globally, placing considerable stress on national economies and challenges for public policies due to the unjustifiable preventable deaths [1–2]. Projections indicate that two of the leading injury causes of deaths will climb in rank by 2040; with road traffic injuries anticipated as the eighth leading and self-harm the 11^th^ leading cause of death [3]. A key strategy to reduce the injury burden calls for the development and use of surveillance systems to manage large sets of data, to record, systematise and monitor these injury trends, and evaluate intervention efforts so as to mitigate risk factors [4]. However, only 34 countries, in high-income contexts such as in America, Europe, and Western Pacific are known to have comprehensive injury mortality surveillance systems [2]. Of concern is that 90% of injury deaths occur in low-to-middle income countries (LMICs) 2. Despite technological developments, the process of surveillance development and use has been globally slow in LMICs [4–5]. This has compounded the paucity of comparable injury data from LMICs, with targets typically monitored by (inter)national agencies that use estimates based on periodic and often more restricted and sampled population data [6].

National injury mortality surveillance is critical to monitor changes in a population’s injury outcomes to inform relevant injury prevention responses. This is particularly the case in South Africa (SA) where the injury burden is nearly twice the global rate and where injury is the fourth leading cause of death [4, 7]. The reported national age-standardised mortality rate for SA is 100.3 per 100 000 population, with homicide the leading manner of injury death at 35.6%, followed by road traffic injury at 27.1% of injury deaths [8]. The National Development Plan (NDP) adopted by SA envisioned a 50% reduction in injuries and violence within its ‘Health Care for All’ objective [9]. However, SA vital statistics are considered inadequate to track progress to 2030 Sustainable Development Goals (with which the NDP is closely aligned) [8]. This is due to underreporting, poor quality, misclassification, and incompleteness of data [10].

Injury surveillance is characterised as the ongoing collection, analysis and interpretation of data needed for controlling public health practice [11]. This may involve either manual (i.e. recording data on paper) or electronic (i.e. the use of electronic systems to collect and store data) data management practices [12]. With the rapid increase of data volumes, public health agencies have moved towards automated forms of collecting and storing injury mortality data. The management of larger injury datasets however expresses the need for information systems to ensure the safety, quality, timeliness of data, management of errors, reducing time of documentation and the need to link various injury data sources [4, 13]. There may be barriers to such expanded surveillance system implementation, including the absence of local investment, and difficulties in standardisation and institutionalisation of such systems [14].

Whilst it is acknowledged that the management of such datasets is a difficult process, many researchers believe that the benefits outweigh the challenges [15]. Currently, there is no international standard for the development of such systems, especially for injury mortality, although data collection may have been advanced for other country-specific priorities as was recently demonstrated in response to the COVID 19 pandemic [4]. There have been reports on outcome variations of the manual and electronic systems. For example, an out-of-hospital clinic study suggested electronic data processing as superior to manual data processing in case identification, whereas manual processing superseded in particular data quality aspects (e.g., reducing missing outcomes) [16]. Thus, both strategies may be pertinent to maximising the value of quality data. Regular evaluations of surveillance systems are pivotal to strengthening surveillance capacity, performance, and cost effectiveness, and to determine whether it is delivering on its objectives. In South Africa, the National Injury Mortality Surveillance System (NIMSS) is an injury mortality platform, currently focused in Mpumalanga and using both manual and electronic web-based data collection systems [17]. Though Forensic Pathology Service (FPS) staff have been trained in both methods, FPS facilities may employ the manual system, if their facilities are unable to support the automated, web-based version of the NIMSS in which case collected data will be physically documented and subsequently electronically captured. The NIMSS provides both the system formats and the data for accurate and timely information on the incidence, causes and circumstances of injury. This study provides a qualitative exploration of FPS staff perceptions of the implementation barriers and facilitators of manual- and electronic injury mortality surveillance system (also eNIMSS) methods in South Africa. More specifically, the study seeks: (a) to determine the FPS staff perceptions of the manual and electronic systems to deliver- on the core characteristics required by a surveillance system to meet its objectives; and (b) to determine the user preferences as regards these surveillance methods.

## Methodology

### Study design

A qualitative research design was employed to collect and analyse the data. By using focus group discussions (FGDs) the participants provided richer context to understand how system users interact with the surveillance methodologies based on their context. This will carry important implications for surveillance system refinement.

### Study setting

The NIMSS currently has full coverage for Mpumalanga. Mpumalanga, located within the north-eastern part of SA, has a surface area of 76 495 km^2^ and is home to an estimated 4.5 million people, accounting for 7.8% of the country’s population [18]. The province is characterised as semi-rural but also hosts large petrochemical- and manufacturing industries. There are three districts within the province, namely, Ehlanzeni District (housing 40.5% of provincial population), Nkangala District (housing 33.3%) and Gert Sibande District (housing 26.2%). A total of 21 FPS laboratories service the area, and provide professional, medico-legal investigation services of all deaths in the province that are considered ‘non- natural’, including those due to injury. The FPS facilities vary in size across the province and generally comprised of a reception area where families wait, office space, a refrigeration room and an autopsy room. Further, as the province is generally considered rural many facilities do not have access a stable internet infrastructure.

### Population and sampling

FPS staff, namely the district coordinators, FPS managers, and FPS non-management staff (i.e. forensic pathology officers or administrators), formed part of the NIMSS training and surveillance implementation. The training provided background to injury mortality surveillance globally and locally, the conceptualisation of NIMSS, data cleaning and validation protocols, and practical and interactive sessions to work with both the manual and e-system. Once FPS staff completed their training, they then captured information from the Death Register [DR] (including injury and death information extracted from post mortem case files) onto the NIMSS system (manual or electronic depending on the facility preference).

All the FPS staff who received training were invited to participate in the FGD. Out of the 63 trained staff, three passed away during the data collection period and twelve participants attended to work priorities. Through purposive sampling, 47 FPS staff (24 data capturers, 20 facility managers, and 3 district coordinators) were recruited as part of a formative evaluation from November 2019 to November 2021. Participants were employed within their current job for two to 21 years. Their ages ranged from 29 to 59 years, comprising 31 males and 16 females. Four FGDs were conducted at a central conference venue per district, two in 2019 (i.e. with exposure to the manual but before implementation of the e-system) and a follow-up in 2021 (after e-system exposure) with the same participants. The same group of participants participated during the pre- and post-assessment as per their district. All participants received the same exposure to training and were provided the same technical support during implementation. The management (i.e. district coordinator and facility managers) and non-management team (i.e. NIMSS data capturers) were interviewed separately to account for variation in implementation process measures. FGDs were approximately one and a half hours to two and a half hours long and influenced by data saturation per question. There were approximately eight participants per FGD. FPS staff travelled to a central location within their respective district where written informed consent was provided in their preferred language. Prior to the FGD, all participants were provided with a telephonic call to confirm their interest in the FGD and verbal informed consent occurred so that they were aware about their rights as participants and the expectations of FGD. Participation was voluntary. The FGDs were facilitated in English and co-facilitated by the research team and made up of a male and female member. All facilitators were trained in research and either had their Masters degree or in the process of its completion. Given that English was not the participants first language three local vernacular research study team members served as translators during the FGD.

### Data collection schedule

As part of the semi-structured interview schedule (see Appendix A), key questions prompted participants to “tell us more about who was involved in gathering data on NIMSS”; to describe how the “data collection and capturing procedure be improved”; to identify how “data queries be improved”; to “describe the resources needed to operate NIMSS”; to describe “the benefits of implementing NIMSS”; to describe “the challenges of implementing NIMSS”; identify the type of support needed to maintain the functioning of the system”; to describe their preferred data capturing methodology” and to provide “recommendations for system improvements”. These questions aimed to describe participants’ experience with training and implementation of the surveillance system. The NIMSS research team which included all three authors facilitated and co-facilitated the FGDs. The co-facilitator took field notes during the FGD. Debriefing sessions were held with the research team to review the research process and reflect on the FGD sessions. The FGD interview schedule was developed by two senior researchers using the WHO surveillance attributes as a framework; and it was piloted on two occasions, namely with a trained cohort of five data collectors who have a tertiary level diploma, and two students who held their Honours degrees. Feedback sessions followed the training to improve parts and wording that needed amendment. Half of the team speak in the vernacular where English is their second language. Their primary language being either Afrikaans, Xhosa, siSwati, and/or isiZulu. The language distribution is a reflection of our study sample too. The one senior researcher served as main facilitator and had provided training when piloting the tool.

### Data analysis

The FGDs were recorded and transcribed verbatim by professional transcribers using a standardised transcription protocol. Transcripts were reviewed for accuracy and completeness by the research team before analysis. Following data cleaning and de-identification of transcripts, the transcripts were imported into a qualitative data analysis software, namely Atlas TI, for data management and the coding of themes. Data was analysed by two analysts independently who familiarised themselves with the data and coded the first FGD to note commonly occurring patterns and generate initial codes using Braun and Clarke’s thematic analysis [19]. Following coding of all transcripts, the two coders met to review their codes and search for additional themes to generate a code book that conceptualised the themes. Themes were revisited and rereviewed in context of the transcripts. Key themes were aligned to the WHO attributes of implementation simplicity (i.e. the system structure and how easy it is to implement the system), acceptability (i.e. stakeholder’s willingness to execute the system as noted by stakeholder’s participation in both the identification and reporting of cases), timeliness (i.e. the extent to which expected reports were submitted within expected timeliness), flexibility (i.e. the system’s ability to adjust to changing needs including additional data being collected), data quality (i.e. the validity of captured data and completeness of content for reporting), and stability (i.e. the system being operational as needed and the system’s ability to manage, collect and generate data without failure) [20]. The sub themes were continually revisited and confirmed with a third researcher. While the WHO surveillance attributes served as the broader framework for themes, the sub themes were formulated through inductive analyses.

### Trustworthiness of data

The trustworthiness of the data is demonstrated along four key criteria. Firstly, in terms of *credibility*, the tool was piloted to consider whether the language and its content were accessible to all participants, i.e. first and second language English speakers. Debriefing meetings occurred following training and piloting of the tool and to consider whether the questions respond to the research questions and study objectives. Further, the entire research team undertook data collection activities at the FPS facilities per district to understand the context of the work and to promote their skills for improved job performance. Field notes were collected during the FGD and stored for reference purposes, alongside transcribed interviews. Secondly, in terms of its *dependability*, the research team received capacitation on the data collection protocols and they were given the opportunity to serve as facilitator as well during the pilot testing of the tool. The coding accuracy was tested when the researchers initially coded the transcripts independently to search for commonly emerging patterns. Also, the participants were provided feedback on the pre-assessment’s key findings and it was reflected on during the post-assessment; thereby corroborating what they had previously reported. All the participants established a relationship with the research team prior the pre-assessment phase due to field engagement meetings and training provided on the system. This provided the participants with comfort to speak more freely as the research team was already exposed to the challenges they faced. Thirdly, for its *transferability*, the same data collection protocol was used in the three districts with the same recruitment procedure of participants. The research team were provided with the data collection package for reference purposes. Detailed notes were kept on the data collection procedure. The researchers reviewed the recordings against the transcript to account for its accuracy and make the needed amendments. Lastly, where *confirmability* is concerned; trustworthiness was strengthened through investigator triangulation where the sub themes were initially independently analysed and thereafter as a collective to co-construct knowledge. To resolve disagreements on the sub themes, discussions ensued to establish agreement on the appropriate changes to be made. This process served to explore the researcher’s multiple interpretations, reducing research bias. The researchers met on numerous occasions to confirm whether the transcripts were coded consistently. Also, multiple quotations are provided by the participants to reduce individual bias.

## Results

The FPS experiences of the six surveillance system attributes will be reported on in order to determine the extent to which the manual and web-based systems met their operational objectives, and the method preferred by the FPS users. Each attribute is further stratified according to the experiences reported (see Table 1 below).

**Table 1:** Themes and sub-themes which emerged from the FGDs

Theme	Sub Theme
**Simplicity**	Implementation of the Surveillance Methodologies
	Processing NIMSS Data
**Acceptability**	FPS Staff Preparedness and Compliance to System Protocols
	Proclivity for a Particular System Method
**Timeliness**	FPS Staff Continual Reprioritisation of Tasks
	Time Spent on Data Capturing and Validation
	Data Analysis and Reporting
**Flexibility**	Transferability of Surveillance Datasets
	Amendments of e-System Variable List and Errors
	Amendments of e-System Variable List and Errors.
**Data Quality**	Validity of Captured Data
	Completeness of Captured Data
	Completeness of Captured Data
**Stability**	Resource constraints influencing NIMSS Surveillance Operations
	Availability of Real Time Data

### Simplicity

While this attribute draws attention to the surveillance system structure and its ease of operation, it also highlighted the FPS staff training, the time spent on data capturing, cleaning, and maintaining the system.

#### Implementation of the surveillance methodologies

Though staff were provided with training pre-NIMSS data capturing, the management and non-management teams both expressed the need for additional computer literacy training during the post-assessment after they had engaged with the system. NIMSS training alone was thus not seen as sufficient, but basic computer training was required as well. This was not limited to the surveillance system training, but indicated more broadly with reference to the entire FPS scope of practice. This was indicated by the older and new staff, and generally, both management and non-management teams did not draw a distinction between the manual and web-based system, as both were reported to require basic computer literacy. A few FPS staff were however not able to comment at all on the e-system as they had poor network coverage which limited if not precluded it’s the latter’s operation.

Participants indicated the following

- “We were not given any training of using a computer. We were just hired as admin, but you don’t go anywhere else to be trained to do that. You just do what you are told to do but there is no formal training” – (Non-management, Gert Sibande District, 2021).
- “It’s a skill that you need as long as you use a PC (personal computer); that’s a skill that you need for e-NIMSS. …if you are computer literate you are able to work but if you are not, it will be a challenge” – (Non-management, Nkangala District, 2021).
- “… more training is needed. New employees come in, you train that person, then you find out the document has been corrupted. So, the training will help on how or what to do if the format has been corrupted.” - (Non-management, Gert Sibande District, 2021).

#### Processing NIMSS data

The capture of data on the manual and web-based methods were acknowledged to not be different, as the variable requirements were the same, although an indicated preference for those that could use the eNIMSS lied with its simple interface and ease of access. Participants across management and non-management teams indicated that the time taken to capture data on the e-system was much faster than the manual one, although issues with data synchronisation (i.e. forgetting how to hotspot, poor internet reception/WiFi connection) hindered the timely submission of data. Their views remained unchanged at the pre- and post-assessment phase. Participants indicated the following.

- “…that one [e-system] is much better than the manual one, there is not too much difference, it works easier” – (Management, Nkangala District, 2019).
- “…we are we doing both in our facility, the manual one and the whole pack of the eNIMSS system because we are comfortable with each one.” – (Non-management, Gert Sibande District, 2021).
- “…it will take long because the internet was giving us a challenge, …a line would come and go, so it would be slow for the whole day; even at night you have to go to the office.” – (Non-management, Gert Sibande District, 2021).

The automated internal validation function on the e-systems was considered a benefit. However, further cleaning of datasets to promote quality and completeness of data was often problematied. Since not all information fields were prompted for capturing this resulted in missing information and data queries and may have undermined the oversight of potential validation errors. This e-system process proved to be cumbersome, with participants reporting the following:

- “… I am trying to cut down on the issue of her calling and sitting [me down] for hours and hours…so with the eNIMSS if she is able to pick it up [the data] then and there, then maybe we can fix it by that time then you know that you are done.” – (Non-management, Ehlanzeni District, 2021).
- “…when she [ NIMSS trained researcher] calls, she will tell us that this side and that side there is something wrong, can you please rectify this. Then we have to go to the files because some of the mistakes that happen there is because of us.” - (Non-management, Ehlanzeni District, 2021).

### Acceptability

FPS staff willingness to implement the manual and e-system when it came to the identification and reporting of case information were key markers of their acceptability of the system and its protocols. Key subthemes exemplified the FPS staff adaptation through their local preparedness and compliance to system protocols, and proclivity for a particular method.

#### FPS staff preparedness and compliance to system protocols

While FPS staff, across the management and non-management teams, generally expressed acceptance and ownership of the system as part of their workload and responsibility, they too adopted local measures to ensure continuation of the capturing load should particular staff be unavailable and work collaboratively. Both management and non-management expressed willingness to assist with data queries to improve the quality of data. Participants indicated the following:

- “NIMSS is part of the job we are doing; [such as] collection, dissection, handover the body, paperwork and capturing.” – (Management, Gert Sibande District, 2019).
- “…every one must be familiar with the system because if one is on leave then others in office must carry on, because everyone at the office will know what to do.” – (Management, Gert Sibande District, 2019).
- “… in our facility we are only 3 FPOs [forensic pathology officers], so they are all involved in the capturing; but there is one, he is the master so we support him.” - (Management, Ehlanzeni District,, 2021).

#### Proclivity for a particular system method

The manual system was preferred as it allowed participants to decide on the data capturing process, and it eased the transference of data. Challenges with the e-system that influenced limited engagement with the method included poor or limited network infrastructure which was required for data synchronisation. This concern was raised even before the implementation of the e-system across the management and non-management teams. The participants indicated the following.

- “…with the manual NIMSS… you can capture… how many cases you want to or which fields you want to, you can choose” – (Non-management, Gert Sibande District, 2021).
- “Even if somebody [manually] captured then left it unfinished you can just take the DR book and finish everything there. You don’t need anybody to explain what you must do. The column tells you, you need this, you put in; as long as the DR book is done properly” – (Non-management, Gert Sibande District, 2021).
- “The [manually filled in] spreadsheet, the easiest one that one” – (Non-management, Gert Sibande District, 2021).

Other cited challenges from management and non-management FGDs included the editing, storing, backing up and dissemination of data. For instance, once a case is submitted on the e-system the user is unable to update the case information. Further, there is concern for loss of data should timely synchronisation not occur. Participants indicated the following:

- “You know the risk of loss of documents and damage of documents is quite high and computers crash every now and then and they can lose information”- (Management, Gert Sibande District, 2021).
- “I submitted the [e-system] file with an error, and then on our side I won’t be able to rectify that mistake” – (Non-management, Gert Sibande District, 2021).

### Timeliness

This attribute demonstrates how the expected surveillance report targets were delivered on within the agreed period. Key factors which influenced this attribute included FPS staff reprioritisation of tasks, the time FPS staff spent on capturing and validating data, and responding to report requests.

#### FPS staff continual reprioritisation of tasks

Data capturing and validation is a time-driven process, however, more often than not, continual delays occurred due to competing work demands. Often, they reprioritised their tasks which affected the time available to complete surveillance activities. Recommendation made by one manager was for an embedded notification system to automatically remind FPS staff at regular intervals of data capturing timelines. Other management staff asserted that FPS facility level teams needed to self-manage with the respective facility manager providing oversight. Thus, communication was cited as key. Participants reported the following:

- “It does matter because you get tired of looking here and going back, then you say ‘ah let me just rest’ then you forget about… tomorrow then you are busy then you can’t do it again.“- (Non-management, Ehlanzeni District, 2021).
- “…from a monthly basis if there was something like…an alarm; it goes to your phone. It reminds you as a facility manager that you are behind for February. It’s reminding you because we are always busy. We may not know that we are behind and by looking into the progress every month it’s easy. These things will assist us.” - (Management, Gert Sibande District, 2021).
- “…your job description says, ‘ensure that data are captured on the NIMSS system on a weekly basis’. …at least choose a day like a Friday when things have cooled off, and work on the cases you were busy with that particular week. You don’t wait for the end of the month. You don’t wait for the end of the year. Already you have a reminder that says there’s a [staff data capturing] schedule. You as a facility manager check on the system whether data was captured the previous week. It’s just a matter of interaction and communication.” - (Management, Gert Sibande District, 2021).

#### Time spent on data capturing and validation

Time taken to capture data varied, and, more often than not, occurred alongside competing tasks (i.e. attending to families of the deceased, collecting a body, assisting with autopsies). Irrespective of the surveillance method used, a contributor to delays in data capturing was the time needed to calculate the deceased’s age from the date of birth record or the ID/passport number. This did not differ for the FPS groups. Data capturing was facilitated when all of the information needed was immediately available at the time of capturing and validation. Participants indicated the following:

- “The issue of speed of capturing because most facilities have people that are not quick at operating a computer as us young ones. I have that luxury of operating quick so, I capture five cases in 15 minutes, he will maybe capture three.” – (Non-management, Ehlanzeni District, 2021).
- “It depends on the information we get from the DR, if the information is there then it becomes easier. It won’t take that much [time], but most of the time the information is missing so it takes time to check in the file. If we don’t find it in the file it’s another challenge, you must find whoever collected the body so that you get the information that you want…it takes time.” – (Non-management, Nkangala District, 2021).
- “If the DR book is not correct nothing will be fine. Then you have to phone the investigating officer and get information.” – (Non-management, Gert Sibande District, 2019).

#### Data analysis and reporting

While participants recognised the benefits of using NIMSS to assist with their monthly reporting requirements and responding to data requests, they experienced challenges with the adoption of the data extraction and reporting component. When FPS staff responded to provincial or parliamentary data requests, management and non-management staff had to undertake a physical count of the caseload which proved time consuming and mentally draining. This manner of responding to data requests led to the underreporting of case information as the primary resources has not been validated. Participants reported the following.

- “…they have to go and open up the DR books. It takes them the whole day to finish that exercise while I’m waiting at the district office so that I can submit to national. By the time [I want to submit then] there’s nobody in the offices” – (Management, Gert Sibande District, 2021).
- “We send out [the data request] to our districts [coordinators] and then they must ask the facility managers to assist. You know you have to go count if you don’t have that information readily available. So, like she said for your current month unfortunately you will have to count but because you still didn’t have your data captured.” – (Management, Ehlanzeni District, 2021).
- “…because you are doing them [the report] manually, sometimes there is inconsistencies in terms of quantities, accuracy, and all of that. But with the NIMSS data you can draw a report of every case that you’ve done in the facility for that particular month. It’s a tool that is made available to all of us to be able to access information without any hassles. Now look how long it takes you to prepare your monthly report at the end of the month.” – (Management, Gert Sibande District, 2021).

### Flexibility

The ability of the manual and e-systems to adapt to the changing FPS operational conditions and project needs with minimal cost includes the ability to add or remove data and modify variable names. Key subthemes include the transferability of the NIMSS dataset, and the ease of updating e-system variable lists.

#### Transferability of surveillance datasets

Numerous FPS facilities experienced challenges with internet connections due to poor network or infrastructure. The manual NIMSS was thus considered practical due to its transferability of the Excel document across computer devices. Participants across the management and non-management team had the same experience. Participants indicated the following.

- “…with the issue of sending information to you we are still struggling with our IT [information technologist] here in the district. He always postpones coming and installing the line for us, but he promised that he will come today. Normally we capture the information on our own [DoH] computer, and not the one for NIMSS…then we put [or transfer] it to the [USB] stick and go to our neighbours at the hospital and send it from there.“ - (Management, Gert Sibande District, 2021).
- “It’s not a decision cast in stone, it is dependent on what is available at the time. If the [NIMSS] computer is out of order, the manual [NIMSS] form should be implemented and sent through so that we do not have a backlog as a facility. When we fill in the paper version, we transmit it the MRC office and then they capture it; then we are done with that part.” – (Management, Ehlanzeni District, 2021).

#### Amendments of e-system variable list and errors

A few of the FPS facilities found the e-system variable list as outdated. This was noted as an ever-occurring issue for particular facilities at management and non-management level. For those whose variable list has been updated, amendments were affordable to undertake, in terms of cost, time, and practicality (i.e. less human resource and applied in a short period). Participants indicated the following:

- “…I will rectify it [the data] myself before submission, but the [eNIMSS] system does not allow me. That is not right.” – (Non-management, Gert Sibande District, 2021).
- “…the list has members who are no longer with us and now we’ve got new members who need to capture. New doctors are also not on the system.” (Management, Nkangala District, 2019).
- “The FPO’s that are appearing on that system are old and the new ones that came after [the implementation of the] eNIMSS are not there.” – (Non-management, Nkangala District, 2021).

### Data quality

This attribute evaluates the captured data’s validity, and the completeness of the datasets across the FPS facilities and highlights broader factors which impact the quality of record keeping. Key sub themes include the validity of captured data, and the completeness captured data.

#### Validity of captured data

Data quality remained similar for both methods, despite the e-system’s embedded internal verification. Common errors include capturing the hospital as the place of injury when it was the place of death; capturing a suspected external cause of death instead of postmortem findings; due to delays in ambulance response the actual time of death is delayed; and capturing incorrect ages for suicide deaths. Participants indicated the following.

- “For example, a person is injured in Belfast but taken to a hospital in Witbank. When he dies the accident is recorded in Witbank, even though it happened in Belfast.” – (Management, Nkangala District, 2019).
- “One day…we fetched a deceased at the hospital; he was involved in a car accident but died at hospital. What exactly do we need to put by the scene? You need the hospital or you need the road?” – (Management, Gert Sibande District, 2021).

#### Completeness of captured data

The completeness of captured data speaks to the current state of case information and management of FPS physical resources which influenced NIMSS data collection. Across most FPS facilities, the case information in the DRs were partially or erroneously completed. Missing and unknown data was particularly attributed to documents not completed correctly and comprehensively; missing information in case files; and DRs not being maintained (i.e. torn and missing pages from the register). Further, case information not being fully recorded by the South African Police Service (SAPS) or FPO further increased the risk of missing information. With some cases being transferred between sites, information was lost or misplaced. Managers recollected how transfers from the scene of injury to hospital before the deceased died also led to missing information. Participants indicated the following.

- “…there are challenges like the lack of information. When you call to ask if it was a car or truck that hit the pedestrian, the problem is that we may not have been there [at the injury scene]. We are four FPO’s, and usually the police attend [to the crime scene] and they don’t write which car was involved in the accident. It really creates a challenge and then I have to say it’s an unknown. They need to teach the police how to complete the SAPS 180 because it is there.” – (Non-management, Gert Sibande District, 2021).
- “…we are the last person to be called at the scene. We don’t know what time the person died. So, when you are asking us that question we don’t have any info about that.” – (Non-management, Gert Sibande District, 2021).
- “From my experience we don’t even have archiving, so everything is just in boxes… so sometimes you have to pick up bits and pieces so that is the difficult things… before you can start capturing, you have to get a box… then you find that the things are in the files… stuff is not sitting in order” – (Non-management, Nkangala District, 2021).

### Stability

The stability attribute evaluates whether the NIMSS system is operational as required and its ability to collect, manage, and produce data consistently without disruptions. Key themes include resource contraints influencing surveillance NIMSS operations and availability of real-time data.

#### Resource contraints influencing NIMSS surveillance operations

Poor internet reception or network was cited as a common barrier, by management and non-management staff, to the timely submission of their dataset whether it be the manual or e-system. Participants reported the following.

- “You can’t access the system with the internet. You can’t do anything without the internet. I think that is the main challenge at the facility.”- (Management, Ehlanzeni District, 2021).
- “I will have to buy my data to hotspot the computer or the other one.” (Non-management, Gert Sibande District, 2021).
- “So yes, technical support like how to hotspot, how to wire to connect, connect those system or whatever that PC or the model or whatever to hotspot and be able to connect with the internet” (Non-management, Gert Sibande District, 2021).

#### Availablity of real time data

The management group positioned the importance of data availability and its contribution for its intended purpose, i.e. timely or real time data. Further, the management group highlighted that research was negatively impacted by a backlog of data. Untimely submission of data coupled with contextual constraints caused by Covid-19 lockdown intervention produced problems when it came to working virtually. Participants reported the following.

- “The back log makes it very difficult to address any issue at hand, because it comes as a post factor, when everything is settled. So, we need to work out a plan that is going to assist all of us. We must, as much as possible move with speed to deviate from the current.” - (Management, Ehlanzeni District, 2021).

## Discussion

Injury prevention hinges on the availability of quality, timely and sufficiently detailed information so that health officials and other preventionists may acquire an accurate understanding of injury magnitude and its associated characteristics to enable the formulation of prevention targets [21]. In this study, surveillance system managers and implementers, including both management and non-management teams, were central to the structure, implementation and functioning of the surveillance system. Hence, the insights provided by FPS staff to the system’s performance is considered of particular use to improve the functionality and delivery of the surveillance systems.

Where system simplicity is concerned, this study highlights significant structural and implementational challenges to surveillance operation, specifically surveillance staff capacity and receptivity to training, the user friendliness of the surveillance system, and data synchronisation, with these factors impacting on both simplicity, and in hindsight the acceptability of the system used. While the e-system was experienced as user-friendly due to its user-system interface, the manual system was considered more flexible in that it allowed the data capturers to decide how to capture the date (e.g., column by column or case by case, with data more easily transferrable). While it is established that trained support staff are critical to surveillance systems [5], more often than not as indicated in the current study, trained FPS staff capacity are undermined in settings where there are few trained staff, where facilities are generally understaffed, and where the available staff have multiple duties and cannot be exclusively committed to data management.

Despite these limitations, the surveillance staff reported a high degree of acceptability towards the surveillance system, irrespective of the particular method. The staff’s willingness to engage with surveillance are particularly key indicators of acceptability (i.e., stakeholder’s willingness to execute the system as noted by stakeholder’s participation in both the identification and reporting of cases) [20] as FPS staff demonstrated, through their uptake of surveillance system learnings, through the adoption of the NIMSS protocols and practices through either system as part of their work routine, and their development of a preference for a particular NIMSS methodology. In line with the study findings, it is commonly asserted that acceptability of a system is enhanced by including the system actors, i.e. the forensic pathologists, as part of the development and implementation of the surveillance system [13], as their operational needs are accommodated and experienced as such. Kipsaina, Ozanne-Smith and Routley (2015) encourages the centrality of strategic buy-in from system actors such as the forensic pathologists so that they experience the advantages of completing the data collection forms and come to consider their role as critical to the success of the system and not an externally imposed requirement. In this manner, the standardisation of data and its improved quality is enabled. Thus, it is critical for end-users to accept a system, and their engagement critical from its development to its implementation [22].

Where the system’s flexibility is concerned, implementation constraints affecting system stability included software related challenges and poor geographical network infrastructure. With Mpumalanga generally considered as rural, internet connectivity is a common issue manifest especially in remote locations. Similarly, an evaluation of a public health surveillance system of refugee settlements in Uganda in 2016 and 2019 highlighted challenges with internet connectivity as key external challenges faced by surveillance systems [23]. The importance of this issue is further supported by a multinational African (including Uganda, DRC, Zambia, Nigeria, and Kenya) injury surveillance project whose delivery on monthly electronic data between coordinating centres was affected due to challenges with internet connection and geographic distance, thus contributing to delays and inefficient monitoring of data quality [24]. Consequently, many cases were not captured into that database and underreporting occurred due to technical issues. The manual NIMSS experienced the same challenges, though, to a lesser degree due to the flexibility of capturing cases offline (i.e. either through the manual or eNIMSS system) and transferability of data (i.e. through the manual NIMSS). These experiences demonstrate that despite the movement to digitise data at entry, the manual data collection superseded the e-system as regards flexibility in dataset management. With the ease of transferability in the manual system, the data is simply transferred from one device to another to increase its accessibility to other users. The manual system also displayed greater fluidity in terms of updating the variable list in comparison to the e-system which required the input of software developers to update the data codes. Kipsaina, Ozanne-Smith and Routley (2015) highlights that the accessibility of their injury mortality surveillance system facilitated the ease of cross integration of a hospital system with the mortuaries central system. An evaluation assessing implementation of a standardised injury mortality surveillance system in LMICs settings also reported that surveillance systems were able to operate with minimal resources when the data collection form was user friendly [5].

Where data quality is concerned, issues experienced with data validity are not new to surveillance systems and the current study echoed this highlighting a number of data collection and capturing issues (e.g. data validity and completeness) which impacted the FPS facilities data capturing performance. For instance, earlier experiences on mortality surveillance in South Africa, in 1998 to 1999, reported that mortuaries captured scene of death rather than scene of injury, and medical service areas (i,e. hospital, clinic, community health clinic, day hospital) were recorded as the scene of injury while it was the scene of death [25]. Moreover, this earlier work indicated that updated information may not be recaptured even if it does become available at a later point [25], and this is similar to what has been seen at the Mpumalanga FPS facilities, signalling that inconsistent and untimely updating of facility records may compromise data quality, irrespective of methodology used. This reemphasised the critical role of data validation and the use of multiple sources to verify complete case information timeously. Furthermore, when listing non-specific categories such as “other” as an option under disease classification, it contributed to further complexity within the surveillance system as also indicated elsewhere [26]. Similar findings were found in other investigations [20], with e.g. nearly half of surveillance system experts experiencing challenges with identifying the main and secondary cause of death [26], a concern with the NIMSS data capturing as well. A summary report using data from the CDC’s National Violent Death Reporting System (NVDRS) concerning violence related deaths in the United States [27] noted challenges to its web-based system such as the timeliness, availability and completeness of data due to dependency on various stakeholders (e.g., state health departments, law enforcement) and furthered that investigative reports may not always contain complete incident information [27]. These challenges were experienced by the NIMSS end-users regardless of the method used. Other challenges noted were multiple and differing death classifications for a single death or incident across different documents (e.g., undetermined on the death certificate, unintentional in a police report, and homicide on a post-mortem report), as also indicated in other studies [27]. NIMSS data capturers experienced difficulties when information was not recorded accurately, with the contribution of SAPS officials to the completeness of data further highlighted. Kipsaina, Ozanne-Smith and Routley (2015) thus advised that the buy-in from all relevant stakeholders to data classification and entry was key to the validity of data.

The timeliness of data is generally impacted by processes to confirm its validity and enable the completeness of the data. More often than not, the external cause of death needs be verified with postmortem findings which has implications for real-time reporting of data should autopsies not have been undertaken. The consequent backlog would be further perpetuated due to pending cases needing to be finalised and the external cause and manner of death to be confirmed. Numerous other injury mortality surveillance systems have noted similar challenges when capturing and reporting data [26, 28]. For example, a Zimbabwean evaluation of a maternal mortality surveillance system supportively notes that increased workload, lack of information and insufficient time to compile validated reports contributed to poor reporting timeliness [28]. A New York abortion reporting surveillance system was also delayed due to the untimely completion of questionnaires [29]. Other challenges include the consideration of multiple external causes of injury to describe mechanisms of death; the partial completion of case information when bodies were transferred from mortuaries; and time constraints staff were subjected too due to increasing workloads 5. Other multi-national studies have indicated that while their data capturers require three to four minutes to capture real-time data, challenges occurred with common missing items, such as injury location and type of road user for road traffic deaths [5]. The surveillance system structure and appearance also play a key role in the time required to submit reports as well as receive feedback [26, 30]. Other studies also reported that regular reporting of data is affected due to poor reporting capabilities, and the inability to transfer information [5, 26]. In this study, the manual and electronic systems were both impacted by the challenges discussed above, with timeliness of submission of the eNIMSS further compounded by poor to no internet and software challenges (including data not synchronising). It is also in this instance that the manual system outperformed the e-system in that missing data were minimised as the case information proved more complete, as also indicated in other investigations [16]. The timeliness of quality data remains a key determinant of decision and policy making in public health [31]. With this said, this study demonstrated that the reporting function was underutilised when responding to monthly reporting requirements. The FPS staff experienced challenges with the adoption of the data extraction and reporting component. Alemu et al. (2019) also noted that the Northwest Ethiopia public health surveillance system reported an underutilisation of analysis and interpretation of data, indicating that this was due to poor or no legal enforcement of surveillance activity, a poor supervision system, no incentivisation and no feelings of data ownership [20]. The non-use of the reporting function, despite training may indicate a skills gap in data management practices where the FPS management is concerned and no enforcement to support the use of the function where it is not institutionalised. Alemu and colleagues (2019) further identified a deficiency of capacity building and training as reasons for this too, proposing feedback loops through regular epidemiological bulletins to show trends, progress to reports, or control of outbreaks [20].

This study reports that staff availability influenced the stability of the system; however, others such by Alemu et al. (2019) notes that their surveillance was less affected by inadequate resources and staff turnover. A Maternal and Perinatal Death Surveillance and Response (MPDSR) carried out in Sub-Saharan Africa (Zimbabwe, Nigeria, Tanzania and Rwanda) however, similarly noted that barriers to implementation include limited time, high staff turnover and staff shortages [32] which impacted the availability of real-time data and facility operations. More often than not, the main justification for this dissimilarity is due to local and regional government support for surveillance systems, and FPS surveillance system integration as part of the broader health system. The multinational study (including Uganda, Nigeria, Zambia, Kenya and the Democratic Republic of Congo) by Zavala, Bokongo, John et al. (2008) also demonstrated that the retrospective collection of large datasets was problematic as there was not sufficient time to allow for complete capturing of information.

While the significance of such information for injury prevention interventions are increasingly recognised, the awareness about the magnitude of the problem has remained low in many countries, with prevention initiatives limited or fragmented [33]. In order to address the international burden of injury mortality, an effective standardised system with locally suitable data entry processes, to timeously record, classify and describe injuries are required [11, 22, 34]. Furthermore, the regular evaluation of a surveillance system is also importance, to provide evidence for strengthening surveillance implementation and reporting [26].

### Strengths and limitations

A key strength is that this study draws from three geographically diverse sites, that includes rural and urban Mpumalanga. A piloted standardised FGD interview schedule was utilised and replicated at pre- and post-assessment which served to validate the FPS perceptions across surveillance system attributes at different points i.e. following training and when they had built nearly two years experience of manual and electronic system engagement. While the study is informed by the WHO surveillance system framework, its attributes do not offer a comprehensive account of the operational parameters for each of the attributes to be measured on. Consequently, sub themes and how it is structured in relation to the constructs predominantly occurred through inductive analysis, thereby offering an expanded clarification of the concepts. Overlap exists with these concepts so parameters had to be set for each attribute. In terms of the sample, majority of the participants who received training consented to the pre- and post FGDs.

While all the study participants considered English as their second language and the medium used at the workplace, there may have been further nuances if they had conversed in their primary language. With the diversity of five local languages and its cultural embeddedness it may not have been feasible for the participants to adequately engage with the group. The opportunity to communicate in their local language was made available as the facilitators could converse adequately and all were equally trained in preparation for the FGD. This exploratory study may serve as foundation for future work to further understand how the identified barriers and facilitators influence the strength of surveillance system attributes. The triangulation of quantitative data will add depth to the performance indicator. Furthermore, participant recall or response bias may have resulted in non-disclosure of other pertinent facilitators and/or barriers experienced in the field, and participants knowledge of the system may have influenced their responses. The authors tried to control for researcher bias by including a third author who was not directly exposed and emerged in the fieldwork.

## Conclusion

Although different countries have specific technical and organisational infrastructures in place, it is pertinent to recognise the relevant system requirements according to its setting so that the relevant system actors may respond accordingly to their surveillance system needs. This was key in this evaluation which sought to elicit the FPS experiences with the system methodologies so to improve their prospective engagement with systems such as the NIMSS. This will facilitate the timely and accurate injury mortality information which is vital to inform public policy, and injury control and prevention responses. Such information on mortality rates and trends is required for monitoring population health status and the identification of emerging health priorities, for the formulation of health interventions and policies [35]. Public health surveillance is key to enhancing population health due to its deliverance of data that can drive quality decision making [21]. Irrespective of the system method used, the evaluation of systems may promote system strengthening and support active tracking of injury mortality surveillance that is aligned to the organisation’s operational needs.

### Electronic supplementary material

Below is the link to the electronic supplementary material.

Supplementary Material 1

## Data Availability

The datasets analysed during the current study are not publicly available due to it being confidential but are available from the corresponding author on reasonable request.
