# Supplementary material for: A qualitative exploration of forensic pathology service staff perceptions of the implementation barriers and facilitators of manual- and electronic injury mortality surveillance system methods in South Africa
Source: BMC Public Health. 2023 Nov 28;23:2354. doi: 10.1186/s12889-023-17337-5 (PMC10685614; doi:10.1186/s12889-023-17337-5)
Supplement: Supplementary file 1 — Supplementary Material 1 [file 12889_2023_17337_MOESM1_ESM.docx]

**Appendix A:**

**Focus Group Interview Guide on the FPS users experience of NIMSS methodologies:**

1. Can you tell us more about who was involved in gathering data on NIMSS?
   1. Was the amount of training received adequate?
   2. Do you require any other training?
   3. What skills are most important to equip yourself to work on NIMSS?
   4. Do you use the eNIMSS or manual NIMSS methodology?
   5. Which NIMSS system is suited for your facility?
   6. How much time was spent collecting data?
   7. How much time was spent capturing data?
   8. Which NIMSS method is more feasible?
2. How can the data collection and capturing procedure be improved?
   1. What process do you follow when collect and capture data?
   2. What type of challenges do you experience?
   3. What measures did you put in place to prevent challenges?
3. How can data queries be improved?
4. Can you describe the resources needed to operate NIMSS?
   1. How was the system maintained?
   2. Who was involved in maintaining the system?
5. What are the benefits of implementing NIMSS?
   1. How has the system assisted the FPS facility data processing?
   2. How has it facilitated the overall functioning of the FPS facility in any way?
   3. How has the process facilitated with any output reports?
   4. In which way has the system been suitable for your context?
6. What are the challenges you experience with NIMSS?
   1. What were the problems experienced with manual NIMSS?
   2. What were the problems experienced with eNIMSS?
7. What type of support do you need to maintain the functioning of the system?
   1. Do you need more support from the district FPS manager?
   2. Does the system need to be further integrated as part of the FPS regulations?
   3. Do you require more technical support?
   4. Do you need more support with the functioning and/or security of the computers?
8. What are your other recommendations for system improvements?
